# Metabolome Genome-Wide Association Study Identifies 74 Novel Genomic Regions Influencing Plasma Metabolites Levels

**DOI:** 10.3390/metabo12010061

**Published:** 2022-01-11

**Authors:** Pirro G. Hysi, Massimo Mangino, Paraskevi Christofidou, Mario Falchi, Edward D. Karoly, NIHR Bioresource Investigators, Robert P. Mohney, Ana M. Valdes, Tim D. Spector, Cristina Menni

**Affiliations:** 1 Department of Twin Research and Genetic Epidemiology, King’s College London, London SE1 7EH, UK; pirro.hysi@kcl.ac.uk (P.G.H.); massimo.mangino@kcl.ac.uk (M.M.); paraskevi.christofidou@kcl.ac.uk (P.C.); mario.falchi@kcl.ac.uk (M.F.); ana.valdes@nottingham.ac.uk (A.M.V.); 2 NIHR Biomedical Research Centre at Guy’s and St. Thomas’ Foundation Trust, London SE1 9RT, UK; 3 Discovery and Translational Sciences, Metabolon Inc., Raleigh-Durham, NC 27560, USA; EKaroly@metabolon.com (E.D.K.); rpmohney@gmail.com (R.P.M.); 4 Inflammation, Injury and Recovery Sciences, School of Medicine, University of Nottingham, Nottingham NG5 1PB, UK

**Keywords:** metabolomics, genome-wise association study, bioresource

## Abstract

Metabolites are small products of metabolism that provide a snapshot of the wellbeing of an organism and the mechanisms that control key physiological processes involved in health and disease. Here we report the results of a genome-wide association study of 722 circulating metabolite levels in 8809 subjects of European origin, providing both breadth and depth. These analyses identified 202 unique genomic regions whose variations are associated with the circulating levels of 478 different metabolites. Replication with a subset of 208 metabolites that were available in an independent dataset for a cohort of 1768 European subjects confirmed the robust associations, including 74 novel genomic regions not associated with any metabolites in previous works. This study enhances our knowledge of genetic mechanisms controlling human metabolism. Our findings have major potential for identifying novel targets and developing new therapeutic strategies.

## 1. Introduction

Metabolism denotes the repertoire of biochemical processes that sustain the life of a cell or organism. Metabolites are small molecules that are by-products or end-products of metabolic processes and are potentially important markers for the states of physiological processes underlying homeostasis. Although numerous external factors may affect metabolite levels, such as nutrition [1,2], drugs [3] and the gut microbiome [4], the metabolome is strongly heritable [5] and genetically-driven. Previous genetic association studies have identified genetic variants influencing circulating blood [6,7,8,9,10,11,12,13,14], urinary excreted [15,16,17], fecal [18] and saliva [19] metabolite levels. Knowledge of the mechanisms controlling the human metabolome is key to understanding physiological processes and pathways involved in health and disease. Various factors impede our understanding of the genetic control mechanisms of the human metabolome, including insufficient resolution for the characterization of both genetic and metabolite markers (i.e., limits in genomic coverage and metabotyping), and the statistical power limitations of most available cohorts. Here we report a genome-wide association study (GWAS) of 722 circulating blood metabolite levels using over 10 million Haplotype Reference Consortium-imputed genetic markers from up to 8809 European participants of the NIHR UK Bioresource cohort. To validate the results, we compared these findings with previously published [12] results from a GWAS on the circulating levels of a subset of 201 overlapping metabolites, conducted on 2 million genetic markers from 1768 individuals.

## 2. Results

**Figure 1:**
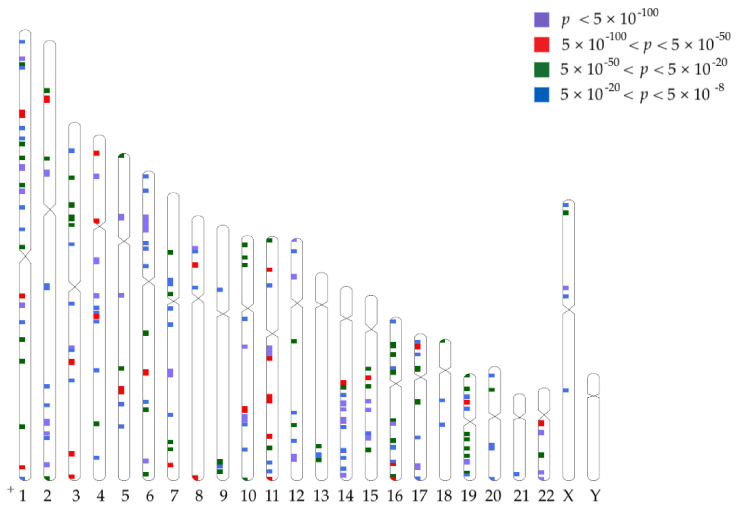
A plot of the locations of the main regions associated with metabolite levels in the discovery cohort.

We conducted GWAS on the plasma levels of 722 metabolites, using over 10 million genetic markers (either directly genotyped or HRC-imputed) as predictors, and we adjusted for age (mean = 48.2 ± 13.46 yrs), sex (M:F = 52.9%:47.1%) and body mass index (BMI) (mean = 27.2 ± 5.4 Kg/m^2^). All analyses had low genomic control factors [20], suggesting no undue inflation or population structure (Table S1). An LD score-based analysis [21] showed that circulating levels of most of the metabolites analyzed had high heritability estimates (Table S2 and Figure S1). Across all analyses, significant associations (*p* < 6.92 × 10^−11^, i.e., conventional GWAS association [22] of 5 × 10^−8,^ adjusted for 722 tests) were found for 152,369 unique genetic markers (Figure 1), which are clustered in 197 unique genomic regions (defined by contiguously associated markers, separated by more than 1 Mbp from other GWAS-associated markers, as recommended previously [23]) located across the genome. Collectively, these markers were associated with the levels of 478 of the metabolites analyzed.

The strongest associations observed were between rs1799958, a seventh exon missense mutation (Gly > Ser) of the ACADS gene, and butyrylcarnitine (*p* = 7.87 × 10^−717^ previously reported [10,24]) and ethylsuccinate (*p* = 1.34 × 10^−962^)—the latter is being reported for the first time. The ACADS gene encodes a human short-chain acyl-CoA dehydrogenase, which catalyzes the initial step of the mitochondrial fatty acid beta-oxidation pathway. Other strong associations were observed between rs2147896 (Met > Thr) within the PYROXD2 gene and with N-methylpipecolate levels (*p* = 4.51 × 10^−946^). This locus has already been reported to be associated with several urinary metabolite levels [16], and with plasma N-methylpipecolate levels [12].

To highlight genotype–metabolite associations that were novel, we compared our findings with the list of known associations between genetic variants and metabolic traits from the GWAS Catalog repository [25]. In total, there were 72 unique genomic regions significantly (*p* < 6.92 × 10^−11^) associated in our analysis with the circulating levels of at least one metabolite which at the time of writing were not listed in the GWAS Catalogue (Table S3). Often, a certain level of metabolite was associated with more than one polymorphism within each region (Table S4).

Among the strongest of the novel associations observed was that between rs1206228892 and imidazole lactate levels (*p* = 4.62 × 10^−186^), and another was that between the rs2042367 variant within the DNAJC16 gene and 4-guanidinobutanoate levels (*p* = 4383 × 10^−165^). SNP rs1801019, a missense variant within the genomic sequence of the UMPS gene, was also strongly associated with orotate levels (*p* = 8.85 × 10^−153^).

Genetic variants associated with metabolites in our analyses displayed significant eQTL effects in different tissues (Table S5), often in several genes. For example, the newly associated rs2042367, located within DNAJC16, has strong eQTL effects over the adjacent CASP9, AGMAT, DNAJC16 and PLEKHM2 genes in the thyroid, pancreatic, esophagus mucosa and skeletal muscle GTEx [26] tissues (*p* = 9.60 × 10 × 10^−24^, *p* = 9.50 × 10 × 10^−20^, *p* = 2.60 × 10 × 10^−12^ and *p* = 6.00 × 10^−18^ respectively). Interestingly, the strongest eQTL effects were observed in central nervous system tissues (Figure S2).

Some of the new variants most strongly associated with metabolites are located within or near genes known to harbor mutations that cause Mendelian disorders of metabolism (Tables S3 and S5). For example, rs41272687, for which we report a novel association with circulating metabolites (*p* = 8.72 × 10^−14^ with 7-HOCA), is a missense mutation in the CYP27A1 gene, whose mutations cause cerebrotendinous xanthomatosis [27]. Similarly, rs1801019, for which we report a new significant association with orotate (*p* = 8.85 × 10^−153^), is a missense variant located within the fourth exon of the UMPS gene, whose mutations cause hereditary orotic aciduria [28].

The associations identified through our analyses showed similarities with genetic associations observed for other traits or diseases. Unsurprisingly, several metabolites were genetically correlated with phenotypic traits correlated with hepatic or renal functions (Table S6). There were also highly statistically significant genetic correlations between levels of metabolites that we analyzed and body mass, height and basal metabolism. These genetic correlations were strong even after linear adjustment for BMI, and remained significant in stratified analyses (Figure S3), which suggests real genetic pleiotropic effects and no confounding. Socio-economic markers, such as those related to educational attainment or deprivation index, were also genetically correlated with the levels of many metabolites, perhaps reflecting the importance of socio-economic differences in nutrition, lifestyle habits and other environmental factors for the entire metabolism.

**Figure 2:**
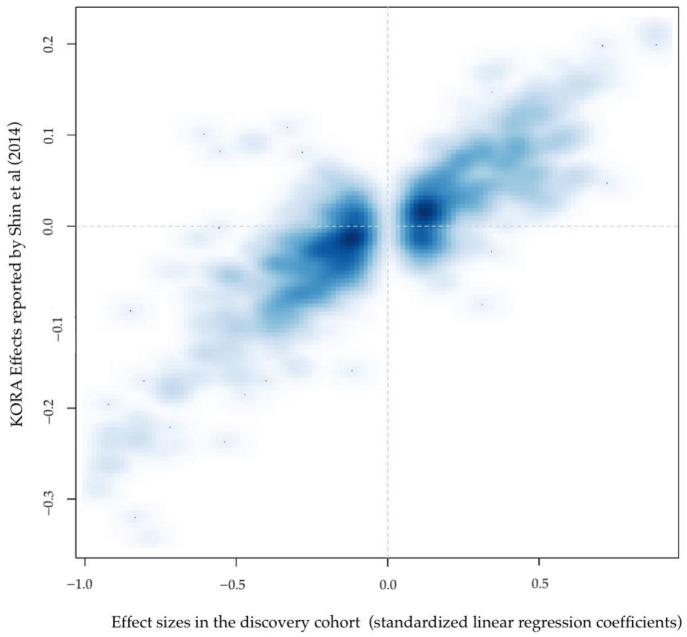
Comparison of the effects of association observed in the NIHR BioResource discovery cohort versus the effects previously reported in the KORA cohort [11].

We compared the results obtained from our GWAS with the summary statistics of the GWAS of the plasma levels of 177 overlapping metabolites from the previously published results on the KORA cohort [12] which are publicly accessible (http://metabolomics.helmholtz-muenchen.de/gwa/si/, accessed on 15 September 2021). Due to the different genetic imputation panels (HRC vs. HapMap2), only a subset of about 2 million genetic markers was available in both datasets. We selected one marker per 92 regions that was associated in our initial discovery GWAS, based on strength of association in the discovery cohort and availability in KORA (Table S7). Nominally significant replication was obtained for 87 out of 102 of the attempted regions, of which 60 remained significant after Bonferroni correction for multiple testing. Beyond statistical significance, the directions of the genetic effects were remarkably consistent in both datasets, for all the other SNPs that were significantly associated with metabolites in the discovery cohort (Figure 2). The meta-analysis of these 201 metabolites identified two additional unique genomic regions for which we report new associations with metabolite levels (Table S8), bringing the total of newly associated metabolically active loci to 74.

To explore the extent to which the effects of the genes associated with the levels of the metabolites, we studied the differences between men and women; we conducted SNP × sex analyses. The interaction associations were different from the marginal effects reported previously. We found little significant genome-wide evidence that the metabolites we studied are differentially regulated in men and women (Table S9). Only two regions, both on chromosome 21, showed significant interactions with sex, although given the large number of metabolites tested, these associations would not remain significant after multiple testing corrections.

## 3. Discussion

To our knowledge, this is the largest scale GWAS of metabolite levels to date. Our genetic investigation of the metabolome benefited both from a large sample size (~9000) and from great breadth of metabolome coverage (722 metabolites). Though previous studies had similar sample sizes to our discovery cohort [7,14], our metabolomic platform measured almost six times as many metabolites. Due to the additional depth and breadth of the analyses, we found 74 novel genomic regions that influence human metabolism.

Some of the associations reported here help shed light on mechanisms of known Mendelian disorder loci. These include the association between 7α-hydroxy-3-oxo-4-cholestenoic acid (7-HOCA) and a cytochrome P450 SNP involved in cerebrotendinous xanthomatosis. The latter is a Mendelian disorder consisting of an accumulation of cholestanol leading to progressive neurological dysfunction, including ataxia, dystonia, dementia, epilepsy, psychiatric disorders, peripheral neuropathy and myopathy [27]. 7-HOCA is a cholestanol metabolite involved in maintaining the integrity of the blood–brain barrier [29,30].

We also report several novel associations with potential clinical relevance. For example, imidazole-lactate is a normally occurring metabolic product of L-histidine transamination, usually excreted in the urine of a number of mammals [31]. L-histidine as an antioxidant is protective against liver fibrosis [32] and against acetaminophen toxicity [33]. The L-histidine degradation pathway also influences the sensitivity of cancer cells to methotrexate and may be related to methotrexate anti-cancer efficacy [34]; therefore, polymorphisms in its degradation pathway may have pharmacogenetic relevance.

Another example is 4-guanidinobutanoate, whose level has already been shown to have very high heritability in mice [34,35]. Although it can be generated as a fungal metabolite, it has been shown to be a gamma-aminobutyric acid (GABA), the chief inhibitory neurotransmitter in the developmentally mature mammalian central nervous system [36]. The relative abundance of this metabolite attenuates the magnitude of meta-amphetamine psychomotor sensitization [36]. The strongest polymorphism associated with levels of this metabolite maps to *DNAJC16*, and also has a strong eQTL effect on that gene in central nervous system tissues.

Interestingly, our work did not find examples of very significant sexual dimorphism in the genetic control of metabolism. It is unclear to what extent this was due to insufficient power to detect genetic interactions or a genuine lack of such interactions in the control of the metabolites that we studied.

There are potential limitations to this work. First, replication was only attempted in about one third of metabolites analyzed during the discovery stage and for a subset of genetic markers; and despite the clear support for our results the replication gave, the lack of direct replication of metabolites unavailable in other platforms was not ideal. Reassuringly, the vast majority of our findings were independently replicated, suggesting the results of this study are robust even more generally. Second, we relied on publicly curated repositories such as the GWAS Catalog for the annotation of our associations. Given the fast pace of publication for GWAS results for a variety of phenotypes, any novel results may not be fully accurate. Genetic associations reported for metabolic traits may not be easy to attribute to specific metabolites, which forms a grey area in which the absolute novelty of certain associations is occasionally not clear-cut. Third, although we would have benefited enormously from the inclusion of individuals of non-European ancestry both in terms of representation of real-world populations and opportunities to better locate association signals, we were unable to analyze a sufficiently large sample of people of other ethnic origins that would have offered sufficient power to improve our study. Fourth, our results further illustrate the fact that genetic annotation of associated variants based on physical distance relative to adjacent genes is often imprecise. They also illustrate the need for a reliable, well curated catalogue of genetic associations with molecular phenotypes, such as metabolites. The latter are often reported in a generic way by the GWAS Catalogue, which also does not take into account several existing results, due their different study designs (for example, when they are generated by analyses that are not classic GWAS), or due to the increased popularity of non-peer reviewed and pre-print publications, which in many cases contain very important information.

Despite limitations, the results of our work shed light on genetic mechanisms controlling human metabolism, which can have many practical implications. Metabolites with significant genetic correlations with BMI can provide useful insights into metabolic dysregulation and obesity. These results may also improve our knowledge of the pharmacokinetics of current and future therapies, as they highlight the complexity and redundancy of metabolic pathways and their genetic control.

More work will be needed in the future to identify variants of functional relevance amid clusters of genetic variants in linkage disequilibrium with each other, and to clarify the roles of the particular genes and transcripts in the control of metabolic processes.

## 4. Materials and Methods

The NIHR BioResource (NBR) Rare Disease Study is a multi-center whole-exome and whole-genome sequencing study including up to 13,600 patients. (http://bioresource.nihr.ac.uk/rare-diseases/rare-diseases/). In this study we analyzed data collected by the NBR. We did not recall any participant for further analysis.

The NBR Rare Diseases study was approved by the East of England Cambridge South national research ethics committee (REC) under reference number 13/EE/0325. The inclusion and exclusion criteria were as described before.

### 4.1. Metabolomic Profiling

Non-targeted metabolite detection and quantification was conducted by the metabolomics provider Metabolon, Inc. (Durham, NC, USA) on fasting plasma samples of 10,654 participants from the UK Bioresource. The metabolomic dataset measured by Metabolon includes 1069 compounds of known structural identity belonging to the following broad categories—amino acids; peptides; carbohydrates; energy intermediates; lipids; nucleotides; cofactors and vitamins; and xenobiotics. A total of 506 compounds of unknown structural identify were also measured.

### 4.2. Quality Control

Sample Handling: Following receipt, samples were inventoried and immediately stored at −80 °C. Each sample received was accessioned into the Metabolon LIMS system and was assigned by the LIMS a unique identifier that was associated with the original source identifier only. This identifier was used to track all sample handling, tasks, results, etc. The samples (and all derived aliquots) were tracked by the LIMS system. All portions of any sample were automatically assigned their own unique identifiers by the LIMS when a new task was created; the relationship of these samples was also tracked. All samples were maintained at −80 °C until processed.

Sample Preparation: Samples were prepared using the automated MicroLab STAR^®^ system from Hamilton Company, headquartered in Reno, Nevada USA). Several recovery standards were added prior to the first step in the extraction process for QC purposes. To remove protein; dissociate small molecules bound to protein or trapped in the precipitated protein matrix; and recover chemically diverse metabolites, proteins were precipitated with methanol under vigorous shaking for 2 min (GenoGrinder 2000, Glen Mills, NJ, USA) followed by centrifugation. The resulting extract was divided into five fractions: two for analysis by two separate reverse phase (RP)/UPLC-MS/MS methods with positive ion mode electrospray ionization (ESI), one for analysis by RP/UPLC-MS/MS with negative ion mode ESI, one for analysis by HILIC/UPLC-MS/MS with negative ion mode ESI and one reserved as a backup. Samples were placed briefly on a TurboVap^®^ (Zymark, Biotage acquired Zymark years ago. Biotage is headquartered in Uppsala, Sweden) to remove the organic solvent. The sample extracts were stored overnight under nitrogen before preparation for analysis. QA/QC: Several types of controls were analyzed in concert with the experimental samples: use of a pool of well-characterized human plasma purchased from bioreclamation. served as a technical replicate throughout the dataset; extracted water samples served as process blanks; and a cocktail of QC standards that were carefully chosen not to interfere with the measurement of endogenous compounds were spiked into every analyzed sample, allowing instrument performance monitoring and aiding in chromatographic alignment. Instrument variability was determined by calculating the median relative standard deviations (RSD) for the standards that were added to each sample prior to injection into the mass spectrometers. Overall process variability was determined by calculating the median RSD for all endogenous metabolites (i.e., non-instrument standards) present in 100% of the pooled matrix samples. Experimental samples were randomized across the platform run with QC samples spaced evenly among the injections.

### 4.3. Ultrahigh Performance Liquid Chromatography–Tandem Mass Spectroscopy (UPLC–MS/MS)

All methods utilized a Waters ACQUITY ultra-performance liquid chromatography (UPLC) and a Thermo Scientific (Waltham, MA, USA) QExactive high resolution/accurate mass spectrometer interfaced with a heated electrospray ionization (HESI-II) source and an Orbitrap mass analyzer operated at 35,000 mass resolution. The sample extract was dried and then reconstituted in solvents compatible with each of the four methods. Each reconstitution solvent contained a series of standards at fixed concentrations to ensure injection and chromatographic consistency. One aliquot was analyzed using acidic positive ion conditions, and chromatographically optimized for more hydrophilic compounds. In this method, the extract was gradient eluted from a C18 column (Waters (Milford, MA, USA) UPLC BEH C18, 2.1 × 100 mm, 1.7 µm) using water and methanol, containing 0.05% perfluoropentanoic acid (PFPA) and 0.1% formic acid (FA). Another aliquot was also analyzed using acidic positive ion conditions, however, it was chromatographically optimized for more hydrophobic compounds. In this method, the extract was gradient eluted from the same aforementioned C18 column using methanol, acetonitrile, water, 0.05% PFPA and 0.01% FA and was operated with an overall higher organic content. Another aliquot was analyzed using basic negative ion optimized conditions using a separate dedicated C18 column. The basic extracts were gradient eluted from the column using methanol and water, and 6.5 mM ammonium bicarbonate at pH 8. The fourth aliquot was analyzed via negative ionization following elution from a HILIC column (Waters(Milford, MA, USA) UPLC BEH Amide 2.1 × 150 mm, 1.7 µm) using a gradient consisting of water and acetonitrile with 10 mM ammonium formate, pH 10.8. The MS analysis alternated between MS and data-dependent MSn scans using dynamic exclusion. The scan range varied slighted between methods but covered 70–1000 *m*/*z*.

Raw data files were archived and extracted as described below.

### 4.4. Bioinformatics

The informatics system consisted of four major components—the Laboratory Information Management System (LIMS); the data extraction and peak-identification software; data processing tools for QC and compound identification; and a collection of information interpretation and visualization tools for use by data analysts. The hardware and software foundations for these informatics components were the LAN backbone, and a database server running Oracle 10.2.0.1 Enterprise Edition.

### 4.5. Data Extraction and Compound Identification

Raw data were extracted, peak-identified and QC processed using Metabolon’s hardware and software. These systems are built on a web-service platform utilizing Microsoft’s. NET technologies, which runs on high-performance application servers and fiber channel storage arrays in clusters to provide active failover and load balancing. Compounds were identified by comparisons with library entries of purified standards or recurrent unknown entities. Metabolon maintains a library based on authenticated standards. The retention time/index (RI), mass to charge ratio (*m*/*z*) and chromatographic data (including MS/MS spectral data) are listed for all molecules present in the library. Furthermore, biochemical identifications are based on three criteria: retention index within a narrow RI window of the proposed identification; accurate mass match to the library +/− 10 ppm; and the MS/MS forward and reverse scores between the experimental data and authentic standards. The MS/MS scores are based on a comparison of the ions present in the experimental spectrum to the ions present in the library spectrum. While there may be similarities between these molecules based on single factors, the use of all three data points can be utilized to distinguish and differentiate biochemicals. More than 3300 commercially available purified standard compounds have been acquired and registered in LIMS for analysis on all platforms for determination of their analytical characteristics. Additional mass spectral entries have been created for structurally unnamed biochemicals, which have been identified by virtue of their recurrent nature (both chromatographic and mass spectral). These compounds have the potential to be identified by future acquisition of a matching purified standard or by classical structural analysis. A variety of curation procedures were carried out to ensure that a high-quality dataset was made available for statistical analysis and data interpretation. The QC and curation processes were designed to ensure accurate and consistent identification of true chemical entities, and to remove those representing system artifacts, mis-assignments and background noise. Library matches for each compound were checked for each sample and corrected if necessary.

### 4.6. Data Quality

Instrument variability was determined by calculating the median relative standard deviation (RSD) for the internal standards that were added to each sample prior to injection into the mass spectrometers. Overall process variability was determined by calculating the median RSD for all endogenous metabolites (i.e., non-instrument standards) present in 100% of the MTRX samples, technical replicates of an extensively characterized large pool of human plasma.

### 4.7. Metabolite Quantification and Data Normalization

Relative metabolite levels were quantified using area under the curve. A data normalization step was performed to correct for variation resulting from instrument inter-day tuning differences. In this step, the raw area for each metabolite was divided by the median value for the run-day batch, thereby setting the medians to equal one (1.00) and normalizing each data point proportionately (termed the “block correction”). This preserved the variation between samples but allowed metabolites of widely different raw peak areas to be compared on a similar graphical scale.

### 4.8. Genotyping and Imputation

Genotyping was carried out with high-density array data (Affymetrix UK Biobank Axiom^®^ Array, ThermoFisher Scientific, Waltham, MA, USA). Before imputation, we performed a range of quality control (QC) measures, excluding both samples and SNPs using the following criteria. We excluded samples with: (1) call rate < 98%; (2) heterozygosity across all SNPs ≥ 3 SD from the sample mean; (3) evidence of non-European ancestry as assessed by PCA comparison with HapMap3 populations; (4) observed pairwise IBD probabilities suggestive of sample identity errors. We then excluded SNPs with: (i) Hardy–Weinberg *p*-value < 10^−6^; (ii) minor allele frequency (MAF) < 1; (iii) call rate < 98%. Furthermore, genotype data were checked for accuracy relative to 1000 G inputs prior to imputation. In particular, we used the “HRC/1KG Imputation Preparation and Checking Tool” (version 4.2.8) developed by Will Rayner to identify: (a) incorrect REF/ALT designations; (b) incorrect strand designations; (c) extreme deviations from expected allele frequencies; and (d) palindromic (A/T and G/C) SNPs with allele frequency near 0.5, which are often the sources of imputation errors. For this process we compared the NIH bioresource using the 1000 G Phase 3 reference panel (http://www.well.ox.ac.uk/~wrayner/tools/1000GP_Phase3_combined.legend.gz, accessed on 10 June 2021).

The cleaned/updated binary files (one for each chromosome) generated by this tool were then converted to vcf using PLINK2 (version 1.90b3.38) and uploaded on the Michigan Imputation Server (https://imputationserver.sph.umich.edu. Accessed:10 June 2021) [37] for the imputation stage. Imputations were performed selecting the following options:

- Reference Panel: 1000 G Phase3 v5;
- Phasing: Eagle v2.3 [38] (Autosomal chromosomes);
- Phasing: ShapeIT [39] (X chromosome).

The results that were obtained from analyses are being reported here using the GRCh37/hg19 as a genomic reference.

### 4.9. Kooperative Gesundheitsforschung in der Region Augsburg (KORA)

Individuals from the follow-up study KORA F4 (Cooperative Health Research in the Region of Augsburg) drawn from the general population of the region of Augsburg, Germany [40]. In total, 1768 individuals with fasting serum metabolomic profiles available using the Metabolon platform and GWAS were analyzed. Summary GWAS results for metabolite levels available in this cohort are publicly accessible (http://metabolomics.helmholtz-muenchen.de/gwa/si/, accessed on 15 September 2021).

### 4.10. Statistical Analysis

Metabolite level normalization and correction of batch effects: Metabolite data were day-median normalized and inverse normalized, as the metabolite concentrations were not normally distributed. To avoid spurious false-positive associations due to small sample size, we excluded metabolic traits with more than 20% missing values, leaving for analysis 722 metabolites of known chemical identity.

Significance and multiple test correction: To account for the large number of tests, we used classic Bonferroni correction, in which the threshold of significance (0.05) was divided by the number of tests (n): α = 0.05/n.

Although many of the tests were not independent (the metabolite levels were inter-correlated), during the discovery stage we considered as significant only the associations with *p* < 5 × 10^−8^/722 = 6.92 × 10^−11^. Similar Bonferroni adjustments were applied to the meta-analysis with the KORA cohort but using 177 (the number of metabolites available from both platforms used) as a correction factor.

Linear regression association analyses: Only individuals of full European ancestry (N = 8809) were included in the analyses in the discovery cohort. To verify ancestry, principal component analysis was carried out on all subjects, and those who were non-European or only partially European (i.e., with appreciable non-European ancestry) were removed from further analyses.

To test for association between metabolite levels and genotypes, we built linear regression models where the outcome was defined as the transformed level of each metabolite, predicted by the allele dosage at each polymorphic (MAF > 0.01) genotyped or imputed genetic variant. In addition, analyses were adjusted for age, sex and body mass index (BMI).

All analyses were conducted using the PLINK software (https://www.cog-genomics.org/plink/2.0/, accessed on 10 June 2021).

Ldscore regression-based methods: To distinguish between the effect of polygenicity and those arising from sample stratification or uncontrolled population admixture, we followed previously suggested approaches [41] to calculate the LD score regression intercepts using the program LD Score (https://github.com/bulik/ldsc, accessed on 10 June 2021). The LD Score method allows for a calculation of the proportion of the phenotypic variance explained by the polymorphisms genotyped or imputed. These estimates are typically smaller than estimates derived from twin or family-based modelling. Genetic correlations between metabolite levels that were subject to our analyses, and other complex traits whose summary statistics are publicly available were assessed following previously described methodologies [42], using the program LD Score (https://github.com/bulik/ldsc, accessed on 10 June 2021).

Conditional and joint association analyses using GWAS summary statistics. To identify multiple sources of association within a genomic region, we conducted analyses that approximated true conditional analysis results (i.e., forward step-wise multivariable linear regression models using as predictors SNPs significantly associated with the outcomes) as described before, implemented in GCTA software [43].

### 4.11. Annotations

Gene annotations: We annotated the results to the nearest gene. For this purpose, we obtained the transcription start and end coordinates on the Human GRCh37/hg19 genome build from the UCSC Genome Browser website (http://genome.ucsc.edu/cgi-bin/hgTracks?db=hg19, accessed on 18 September 2021). Only transcripts that were located within 200,000 bp were considered.

Phenotypic annotations: Previous associations between genetic variants of interest and other disease or quantitative phenotypic traits were retrieved by querying the 15 November 2018 version of the GWAS Catalog [25]. Specifically, we compared our findings against GWAS catalog entries that involved levels of any metabolite in blood (plasma, serum or urine).

If no association with any endogenous metabolite level was reported in the GWAS catalog, the association between that locus and the metabolite level that we observed in our analysis was considered novel. We specifically did not consider metabolic products of man-made substances, such as medications or pollutants.

The Online Mendelian Inheritance in Man (OMIM) is a continuously curated catalog of human genes and phenotypic changes their polymorphic forms cause in humans [44]. This catalogue contains a not fully complete, but highly reliable list of gene–phenotype pairs and was used retrieve data that could inform about the functionality of specific genes with particular focus on phenotypic expressions of extremely penetrant mutations. Annotation data were downloaded from http://omim.org/, (accessed on 18 September 2021)

Functional annotation eQTL: The influences of our significant SNPs on the transcription of adjacent genes were assessed in all other tissues available to the GTEx Project and queried in the GTEx Portal (https://www.gtexportal.org/home/, accessed on 18 September 2021).

### 4.12. Meta-Analyses

Random-effects meta-analyses results were also obtained using the GWAMA (Version 2.2.2) software.

## Data Availability

Data is available upon request from the NIHR BioResource https://bioresource.nihr.ac.uk/using-our-bioresource/.

## Supplementary Materials

The following are available online at https://www.mdpi.com/article/10.3390/metabo12010061/s1. Figure S1. Distribution of the estimate heritability values for the 722 metabolites analyzed. The values plotted here are the estimates of the proportion of phenotypic variability that are explained by directly genotyped or imputed SNPs (“SNP chip heritability”), which are always smaller than heritability estimates from twin and other family-based studies. Figure S2. The distribution of the normalized effect size (NES) across a selection of GTEx-available tissues. Only the most significant eQTL effect per associated region (Table S3) was selected. The top two rows were selected on the basis of high NES medians, and the bottom two due to low NES medians. Figure S3. Association of rs72552254 with threonate by BMI Quintile. This example was selected both because it represents an association that is novel and because among all novel results, this metabolite (threonate) showed the strongest genetic correlation with BMI. Each of the observations marked with a blue dot represents the effect size of the rs72552254 on the threonate levels for each quintile (different rows), and error bars represent the standard error (SE) for the effect size estimation. The last row (in red) represents the effect size and SE for all subjects, regardless of the BMI. Table S1. Genomic inflation factors for genome-wide association studies of all metabolites. Table S2. SNP Chip LDscore-based heritability estimates for all the available metabolites. Table S3. Genomic regions and markers most significantly associated with metabolite levels. Table S4. Additional SNPs identified associated with metabolite levels following Co-Jo condtional analyses. Table S5. Summary of the most significant eQTL effects for a selection of variants most significantly associated with metabolite levels. Table S6. Selection of the most significant Ldscore-based genomic correlations between analyzed metabolites and other GWAS traits. Table S7. Concordance of results and replication between the GWAS in the NIHR Bioresource discovery cohort and the KORA cohort. Table S8. Novel genomic regions associated with metabolite levels, identified as a result of a meta-analysis between the NIHR Bioresource and the KORA cohorts. Table S9. Genome-wide significant (*p* < 5 × 10^−8^) results for SNP × Sex interactions.

## References

[1] Sebedio J.L. (2017). Metabolomics, Nutrition, and Potential Biomarkers of Food Quality, Intake, and Health Status. Adv. Food Nutr. Res..

[2] Pallister T., Sharafi M., Lachance G., Pirastu N., Mohney R.P., MacGregor A., Feskens E.J., Duffy V., Spector T.D., Menni C. (2015). Food Preference Patterns in a UK Twin Cohort. Twin. Res. Hum. Genet..

[3] Kohler I., Hankemeier T., Van der Graaf P.H., Knibbe C.A.J., Van Hasselt J.G.C. (2017). Integrating clinical metabolomics-based biomarker discovery and clinical pharmacology to enable precision medicine. Eur. J. Pharm. Sci..

[4] Liu R., Hong J., Xu X., Feng Q., Zhang D., Gu Y., Shi J., Zhao S., Liu W., Wang X. (2017). Gut microbiome and serum metabolome alterations in obesity and after weight-loss intervention. Nat. Med..

[5] Shah S.H., Hauser E.R., Bain J.R., Muehlbauer M.J., Haynes C., Stevens R.D., Wenner B.R., Dowdy Z.E., Granger C.B., Ginsburg G.S. (2009). High heritability of metabolomic profiles in families burdened with premature cardiovascular disease. Mol. Syst. Biol..

[6] Demirkan A., Van Duijn C.M., Ugocsai P., Isaacs A., Pramstaller P.P., Liebisch G., Wilson J.F., Johansson A., Rudan I., Aulchenko Y.S. (2012). Genome-wide association study identifies novel loci associated with circulating phospho- and sphingolipid concentrations. PLoS Genet..

[7] Draisma H.H.M., Pool R., Kobl M., Jansen R., Petersen A.K., Vaarhorst A.A.M., Yet I., Haller T., Demirkan A., Esko T. (2015). Genome-wide association study identifies novel genetic variants contributing to variation in blood metabolite levels. Nat. Commun..

[8] Illig T., Gieger C., Zhai G., Romisch-Margl W., Wang-Sattler R., Prehn C., Altmaier E., Kastenmuller G., Kato B.S., Mewes H.W. (2010). A genome-wide perspective of genetic variation in human metabolism. Nat. Genet..

[9] Kettunen J., Tukiainen T., Sarin A.P., Ortega-Alonso A., Tikkanen E., Lyytikainen L.P., Kangas A.J., Soininen P., Wurtz P., Silander K. (2012). Genome-wide association study identifies multiple loci influencing human serum metabolite levels. Nat. Genet..

[10] Li Y., Sekula P., Wuttke M., Wahrheit J., Hausknecht B., Schultheiss U.T., Gronwald W., Schlosser P., Tucci S., Ekici A.B. (2018). Genome-Wide Association Studies of Metabolites in Patients with CKD Identify Multiple Loci and Illuminate Tubular Transport Mechanisms. J. Am. Soc. Nephrol..

[11] Long T., Hicks M., Yu H.C., Biggs W.H., Kirkness E.F., Menni C., Zierer J., Small K.S., Mangino M., Messier H. (2017). Whole-genome sequencing identifies common-to-rare variants associated with human blood metabolites. Nat. Genet..

[12] Shin S.Y., Fauman E.B., Petersen A.K., Krumsiek J., Santos R., Huang J., Arnold M., Erte I., Forgetta V., Yang T.P. (2014). An atlas of genetic influences on human blood metabolites. Nat. Genet..

[13] Suhre K., Shin S.Y., Petersen A.K., Mohney R.P., Meredith D., Wägele B., Altmaier E., Deloukas P., Erdmann J., Grundberg E. (2011). Human metabolic individuality in biomedical and pharmaceutical research. Nature.

[14] Lotta L.A., Pietzner M., Stewart I.D., Wittemans L.B.L., Li C., Bonelli R., Raffler J., Biggs E.K., Oliver-Williams C., Auyeung V.P.W. (2021). A cross-platform approach identifies genetic regulators of human metabolism and health. Nat. Genet..

[15] Raffler J., Friedrich N., Arnold M., Kacprowski T., Rueedi R., Altmaier E., Bergmann S., Budde K., Gieger C., Homuth G. (2015). Genome-Wide Association Study with Targeted and Non-targeted NMR Metabolomics Identifies 15 Novel Loci of Urinary Human Metabolic Individuality. PLoS Genet..

[16] Rueedi R., Ledda M., Nicholls A.W., Salek R.M., Marques-Vidal P., Morya E., Sameshima K., Montoliu I., Da Silva L., Collino S. (2014). Genome-wide association study of metabolic traits reveals novel gene-metabolite-disease links. PLoS Genet..

[17] Suhre K., Wallaschofski H., Raffler J., Friedrich N., Haring R., Michael K., Wasner C., Krebs A., Kronenberg F., Chang D. (2011). A genome-wide association study of metabolic traits in human urine. Nat. Genet..

[18] Zierer J., Jackson M.A., Kastenmüller G., Mangino M., Long T., Telenti A., Mohney R.P., Small K.S., Bell J.T., Steves C.J. (2018). The fecal metabolome as a functional readout of the gut microbiome. Nat. Genet..

[19] Nag A., Kurushima Y., Bowyer R.C.E., Wells P.M., Weiss S., Pietzner M., Kocher T., Raffler J., Völker U., Mangino M. (2020). Genome-wide scan identifies novel genetic loci regulating salivary metabolite levels. Hum. Mol. Genet..

[20] Devlin B., Roeder K. (1999). Genomic control for association studies. Biometrics.

[21] Finucane H.K., Bulik-Sullivan B., Gusev A., Trynka G., Reshef Y., Loh P.R., Anttila V., Xu H., Zang C., Farh K. (2015). Partitioning heritability by functional annotation using genome-wide association summary statistics. Nat. Genet..

[22] Pe’er I., Yelensky R., Altshuler D., Daly M.J. (2008). Estimation of the multiple testing burden for genomewide association studies of nearly all common variants. Genet. Epidemiol..

[23] Wood A.R., Esko T., Yang J., Vedantam S., Pers T.H., Gustafsson S., Chu A.Y., Estrada K., Luan J., Kutalik Z. (2014). Defining the role of common variation in the genomic and biological architecture of adult human height. Nat. Genet..

[24] Pedersen C.B., Kolvraa S., Kolvraa A., Stenbroen V., Kjeldsen M., Ensenauer R., Tein I., Matern D., Rinaldo P., Vianey-Saban C. (2008). The ACADS gene variation spectrum in 114 patients with short-chain acyl-CoA dehydrogenase (SCAD) deficiency is dominated by missense variations leading to protein misfolding at the cellular level. Hum. Genet..

[25] MacArthur J., Bowler E., Cerezo M., Gil L., Hall P., Hastings E., Junkins H., McMahon A., Milano A., Morales J. (2017). The new NHGRI-EBI Catalog of published genome-wide association studies (GWAS Catalog). Nucleic Acids Res..

[26] Lonsdale J., Thomas J., Salvatore M., Phillips R., Lo E., Shad S., Hasz R., Walters G., Garcia F., Young N. (2013). The Genotype-Tissue Expression (GTEx) project. Nat. Genet..

[27] Nie S.K., Chen G.Q., Cao X.B., Zhang Y.J. (2014). Cerebrotendinous xanthomatosis: A comprehensive review of pathogenesis, clinical manifestations, diagnosis, and management. Orphanet J. Rare Dis..

[28] Grohmann K., Lauffer H., Lauenstein P., Hoffmann G.F., Seidlitz G. (2015). Hereditary Orotic Aciduria with Epilepsy and without Megaloblastic Anemia. Neuropediatrics.

[29] Saeed A., Floris F., Andersson U., Pikuleva I., Lovgren-Sandblom A., Bjerke M., Paucar M., Wallin A., Svenningsson P., Bjorkhem I. (2014). 7alpha-hydroxy-3-oxo-4-cholestenoic acid in cerebrospinal fluid reflects the integrity of the blood-brain barrier. J. Lipid Res..

[30] Saeed A.A., Edstrom E., Pikuleva I., Eggertsen G., Bjorkhem I. (2017). On the importance of albumin binding for the flux of 7alpha-hydroxy-3-oxo-4-cholestenoic acid in the brain. J. Lipid Res..

[31] Block W.D., Westhoff M.H., Steele B.F. (1967). Histidine metabolism in the human adult: Histidine blood tolerance, and the effect of continued free L-histidine ingestion on the concentration of imidazole compounds in blood and urine. J. Nutr..

[32] El-Batch M., Ibrahim W., Said S. (2011). Effect of histidine on autotaxin activity in experimentally induced liver fibrosis. J. Biochem. Mol. Toxicol..

[33] Yan S.L., Wu S.T., Yin M.C., Chen H.T., Chen H.C. (2009). Protective effects from carnosine and histidine on acetaminophen-induced liver injury. J. Food Sci..

[34] Kanarek N., Keys H.R., Cantor J.R., Lewis C.A., Chan S.H., Kunchok T., Abu-Remaileh M., Freinkman E., Schweitzer L.D., Sabatini D.M. (2018). Histidine catabolism is a major determinant of methotrexate sensitivity. Nature.

[35] Adkins D.E., McClay J.L., Vunck S.A., Batman A.M., Vann R.E., Clark S.L., Souza R.P., Crowley J.J., Sullivan P.F., Van den Oord E.J.C.G. (2013). Behavioral metabolomics analysis identifies novel neurochemical signatures in methamphetamine sensitization. Genes Brain Behav..

[36] Hong H., Fill T., Leadlay P.F. (2013). A Common Origin for Guanidinobutanoate Starter Units in Antifungal Natural Products. Angew. Chem. Int. Edit..

[37] Das S., Forer L., Schonherr S., Sidore C., Locke A.E., Kwong A., Vrieze S.I., Chew E.Y., Levy S., McGue M. (2016). Next-generation genotype imputation service and methods. Nat Genet.

[38] Loh P.O., Danecek P., Palamara P.F., Fuchsberger C., Reshef Y.A., Finucane H.K., Schoenherr S., Forer L., McCarthy S., Abecasis G.R. (2016). Reference-based phasing using the Haplotype Reference Consortium panel. Nat. Genet..

[39] Delaneau O., Howie B., Cox A.J., Zagury J.F., Marchini J. (2013). Haplotype estimation using sequencing reads. Am J Hum Genet.

[40] Wichmann H.E., Gieger C., Illig T., for the MONICA/KORA Study Group (2005). KORA-gen-resource for population genetics, controls and a broad spectrum of disease phenotypes. Gesundheitswesen.

[41] Bulik-Sullivan B.K., Loh P.R., Finucane H.K., Ripke S., Yang J., Patterson N., Daly M.J., Price A.L., Neale B.M., Schizophrenia Working Group of the Psychiatric Genomics Consortium (2015). LD Score regression distinguishes confounding from polygenicity in genome-wide association studies. Nat. Genet..

[42] Bulik-Sullivan B., Finucane H.K., Anttila V., Gusev A., Day F.R., Loh P.R., Duncan L., ReproGen Consortium, Psychiatric Genomics Consortium, Genetic Consortium for Anorexia Nervosa of the Wellcome Trust Case Control Consortium (2015). An atlas of genetic correlations across human diseases and traits. Nat. Genet..

[43] Yang J., Ferreira T., Morris A.P., Medland S.E., Madden P.A., Heath A.C., Martin N.G., Montgomery G.W., GIANT Consortium, DIAGRAM Consortium (2012). Conditional and joint multiple-SNP analysis of GWAS summary statistics identifies additional variants influencing complex traits. Nat. Genet..

[44] McKusick V.A. (2007). Mendelian Inheritance in Man and its online version, OMIM. Am. J. Hum. Genet..

