# Supplementary material for: Metabolome Genome-Wide Association Study Identifies 74 Novel Genomic Regions Influencing Plasma Metabolites Levels
Source: Metabolites. 2022 Jan 11;12(1):61. doi: 10.3390/metabo12010061 (PMC8777659; doi:10.3390/metabo12010061)

Figure S1. Distribution of the estimate heritability values for the 722 metabolites analyzed. The values plotted here are the estimates of the proportion of phenotypic variability that are explained by directly genotyped or imputed SNPs (“SNP chip heritability”), which are always smaller than heritability estimates from twin and other family-based studies.

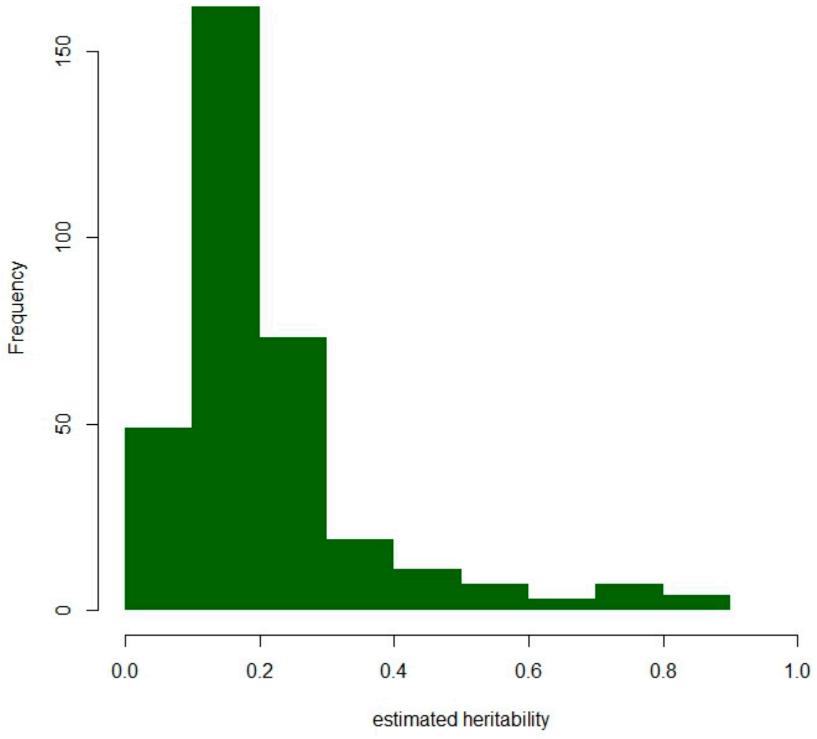

Supplement: Supplementary file 1 [file metabolites-12-00061-s001.zip › Figure S1.pdf]
