# Supplementary material for: Metabolome Genome-Wide Association Study Identifies 74 Novel Genomic Regions Influencing Plasma Metabolites Levels
Source: Metabolites. 2022 Jan 11;12(1):61. doi: 10.3390/metabo12010061 (PMC8777659; doi:10.3390/metabo12010061)

**Figure S2**. The distribution of the normalized effect size (NES) across a selection of GTEx-available tissues. Only the most significant eQTL effect per associated region (Supplementary Table 3) was selected. The top two rows are selected on the basis of a high NES median and the bottom two of a low NES median.

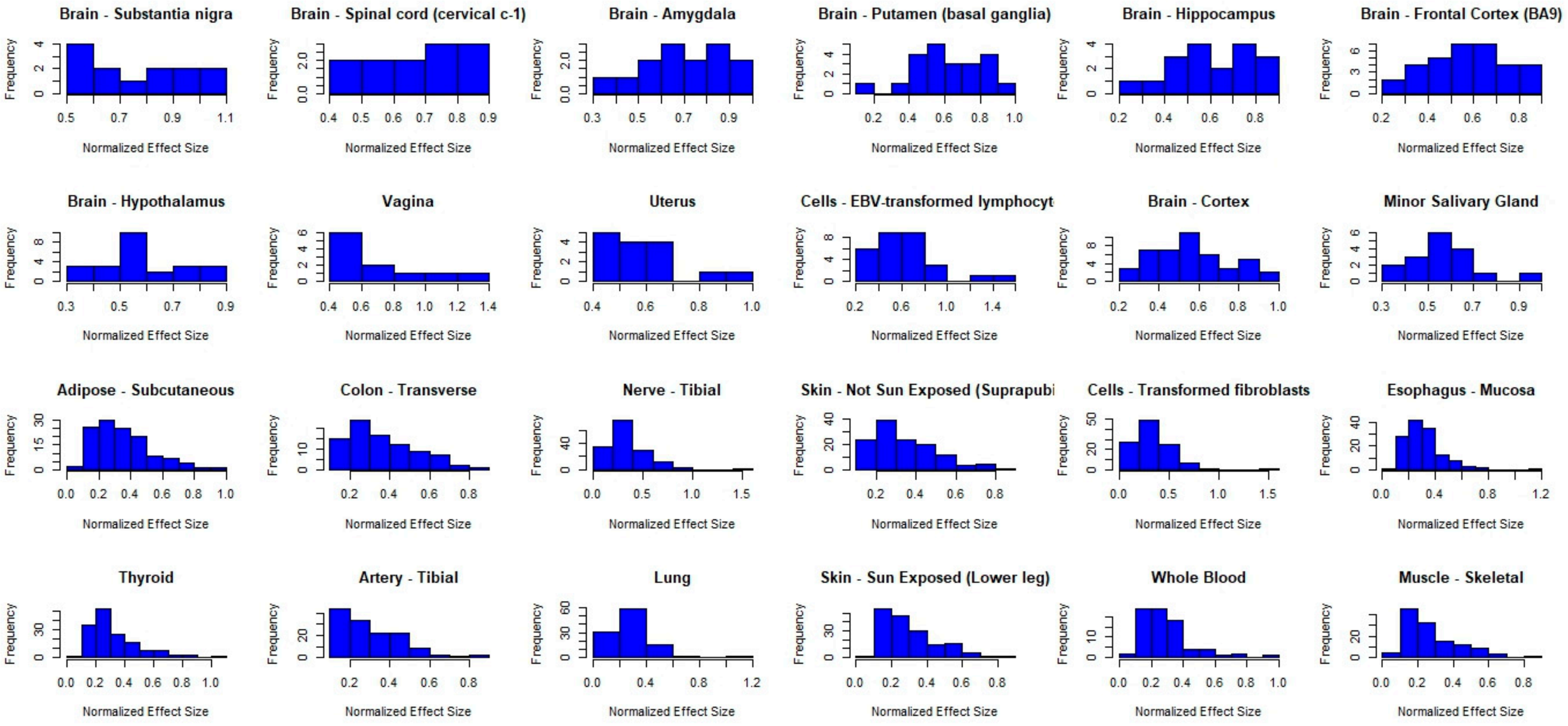

Supplement: Supplementary file 1 [file metabolites-12-00061-s001.zip › Figure S2.pdf]
