# Supplementary material for: Metabolome Genome-Wide Association Study Identifies 74 Novel Genomic Regions Influencing Plasma Metabolites Levels
Source: Metabolites. 2022 Jan 11;12(1):61. doi: 10.3390/metabo12010061 (PMC8777659; doi:10.3390/metabo12010061)

Figure S3. Association of rs72552254 with threonate by BMI Quintile. This example was selected both because it represents an association that is novel and because among all novel results, this metabolite (threonate) showed the strongest genetic correlation with BMI. Each of the observations marked with a blue dot represents the effect size of the rs72552254 on the threonate levels for each quintile (different rows) and error bars represent the standard error (SE) for the effect size estimation. The last row (in red) represents the effect size and SE for all subjects, regardless of the BMI.

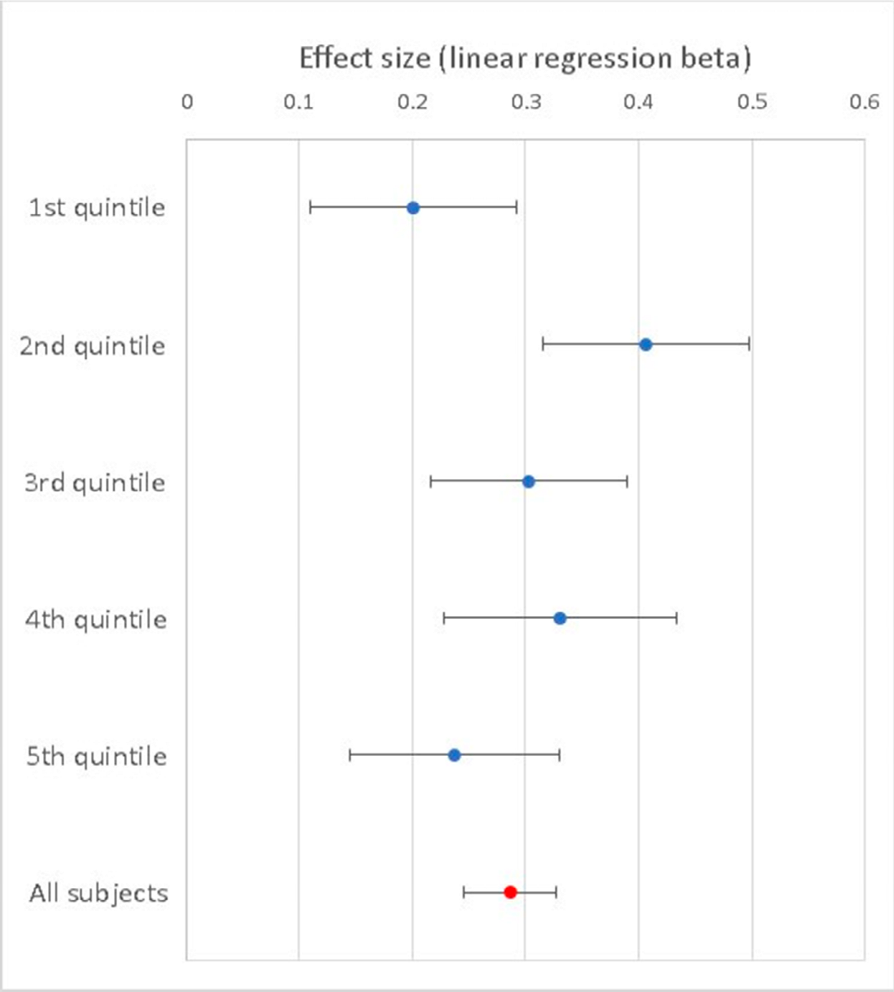

Supplement: Supplementary file 1 [file metabolites-12-00061-s001.zip › Figure S3.pdf]
